# Characteristics Associated With Endowed Chair Titles Among U.S. Academic Ophthalmologists

**DOI:** 10.7759/cureus.85716

**Published:** 2025-06-10

**Authors:** Alex Choi, Alice Haystead, Camryn Thompson, Anita Kundu, Sandra S Stinnett, Lejla Vajzovic, Sharon Fekrat

**Affiliations:** 1 Department of Ophthalmology, Duke University School of Medicine, Durham, USA

**Keywords:** academic ophthalmology, endowed chair, faculty titles, gender disparities, geographic variation, leadership in medicine

## Abstract

Introduction: Over the last several decades, the number of women in ophthalmology has gradually increased. However, studies have demonstrated that far fewer women than men are represented in senior leadership positions. Having an endowed chair facilitates career influence through support for research, service, and teaching activities and is considered one of the most prestigious academic awards that an institution for a faculty member can grant. We explore the relationship between endowed chair status and a variety of characteristics among ophthalmology faculty at 41 major institutionsacross the United States (U.S.).

Methods: All 41 ophthalmology hospitals listed in *US News and World Report's *2022 Best Hospitals for Ophthalmologywere included. Endowed chair status, gender, number of academic titles, *US News *rank of institution, region of the U.S., graduate degree, professor status, and ophthalmic subspecialty were collected using publicly available information. Univariable analysis compared the odds of having an endowed chair for subgroups of the studied variables. Multivariable logistic regression determined the odds of having an endowed chair after controlling for other variable effects.

Results: A total of 860 (38%) women and 1,402 (62%) men ophthalmologists were identified. Of these, 246 (10.9%) carried endowed chair titles, of whom 59 (24%) were women. On univariable analysis, being male (p<0.001), greater number of titles (p<0.001), location in the South (p<0.001), PhD degree (p=0.026), full professor status (p<0.001), and uveitis/retina specialization (p<0.001) had significant associations with endowed chair status. Compared with comprehensive ophthalmologists, most ophthalmic subspecialists had greater odds of endowed chair status (p<0.05). On multivariable analysis, all variables had a significant association with endowed chair status (p<0.001) except for gender (p=0.111).

Conclusion: After controlling for other variables, holding two or more additional titles, location in the South, PhD degree, uveitis/retina specialization, and holding a full professorship were significantly associated with greater odds of holding an endowed chair across U.S. academic ophthalmology institutions. Gender had a significant association with endowed chair status on univariable but not multivariable analysis; however, more men than women had characteristics that this study found to be associated with endowed chair status.

## Introduction

Over the last several decades, the number of women in ophthalmology has gradually increased [1], yet in 2023, only 35%-45% of ophthalmology residents and 25%-30% of ophthalmologists are women, even though half of the United States (U.S.) population is female [2]. There also remains evidence of a gender disparity in annual income even when considerations such as number of hours worked, physician characteristics, and practice profiles are taken into account [3]. Far fewer women than men are represented in senior ophthalmology leadership positions such as editor-in-chief of top journals or president of influential ophthalmology societies [4-6]. Previous studies in medical specialties, including oncology and obstetrics and gynecology, have found significant associations between having an endowed chair and higher professor level as well as greater research productivity for both men and women, as determined by total publications, citations, and H-index [7,8]. Although such associations would be acceptable and expected if higher leadership positions and professorships were solely granted on a merit basis, evidence suggests that this may not always be the case [9]. Women are more likely to bear a disproportionate burden of family responsibilities, workplace sexual harassment, compensation inequities, and lack of access to and support from mentors, among other structural barriers to career advancement [9,10].

Being recognized with an endowed chair typically facilitates career influence, supports effort toward research, service, and teaching activities, and is considered one of the most prestigious academic awards that can be granted by an academic institution for one of its faculty members. To our knowledge, no published studies have examined gender inequities or factors influencing endowed chair status among ophthalmologists affiliated with academic institutions. We explore the relationship between endowed chair status and a variety of characteristics, including gender, among ophthalmology faculty at 41 major institutions across the U.S.

## Materials and methods

**Figure 1:**
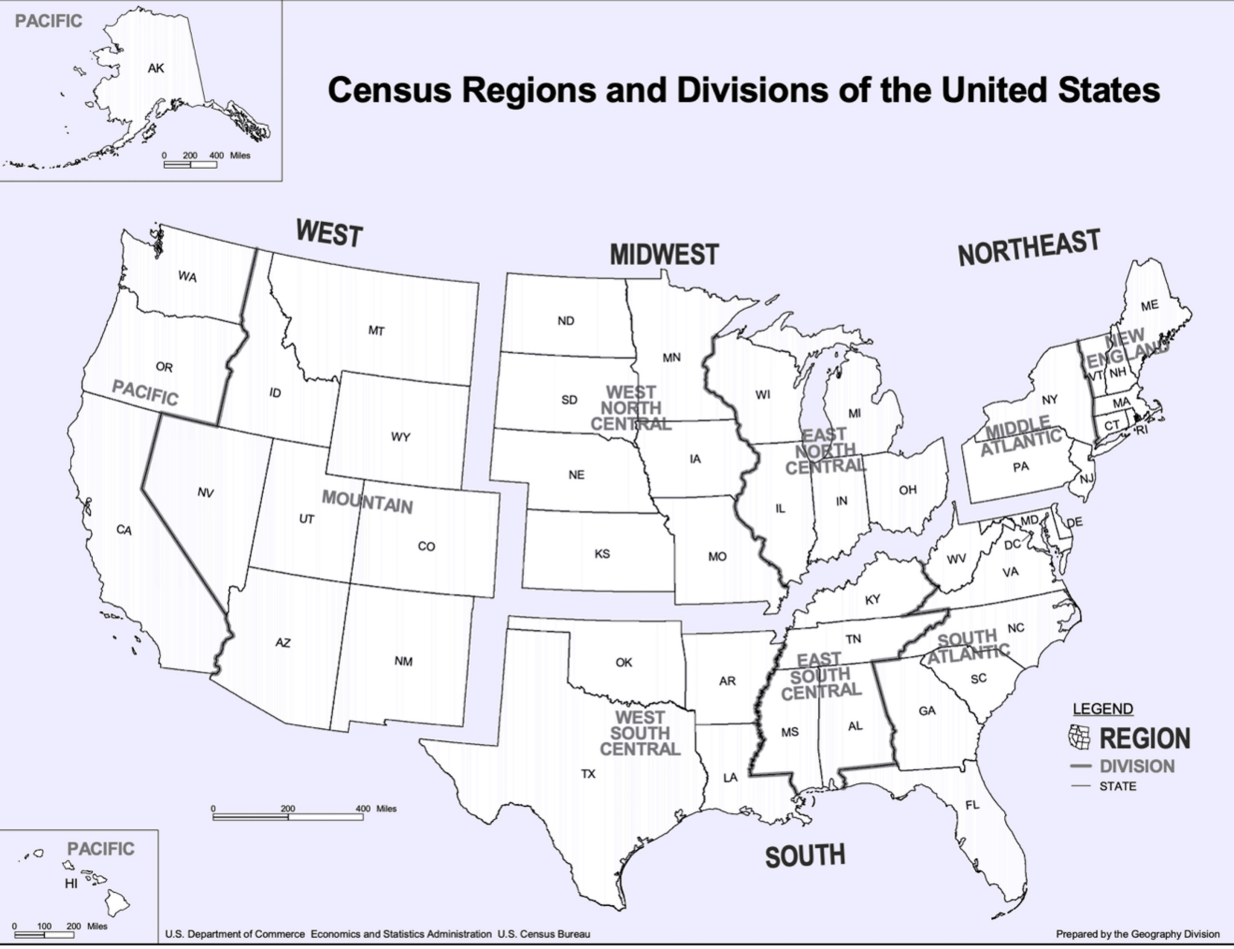
Census Regions and Divisions of the United States Credit: Adapted and modified from the U.S. Census Bureau [12].

This study protocol was approved by the Duke Health Institutional Review Board (Pro00112018) and was adherent to all tenets of the Declaration of Helsinki. All 41 ophthalmology hospitals listed by the *US News and World Report's* 2022 Best Hospitals for Ophthalmology were included [11]. All faculty with graduate degrees, including doctor of medicine (MD), doctor of philosophy (PhD), doctor of osteopathic medicine (DO), and both MD and PhD, on the institutions’ websites as of June 2022, were included in the study. Endowed chair status and faculty member characteristics were collected using publicly available information online, including institutional websites, LinkedIn, ResearchGate, and practice websites. To ensure accuracy, collected data for each faculty member was cross-referenced across multiple sources. Inclusion criteria included faculty members with an MD or PhD degree or both at each of the 41 hospitals. Exclusion criteria included faculty members with solely an optometry degree (OD). For each identified faculty member, the following data were collected: gender, region of the U.S., graduate degree(s), number of academic titles, rank of institution (numbered rank vs. no numbered rank in the 2022 *US News and World Report *list), professor status, and ophthalmic subspecialty. Gender was determined by searching publicly available sources for pronouns such as he/him/his for men and she/her/hers for women. Due to the small sample size of individuals with nonbinary pronouns such as they/them, that cohort was not included in this study. Academic titles included administrative and leadership roles (e.g., Chair, Vice Chair, Director) as listed on institutional profiles. Professor status (assistant, associate, full professor) was recorded as per the most recent academic appointment noted. Subspecialties were assigned based on explicit mention in faculty profiles or practice descriptions (e.g., cornea, glaucoma, comprehensive). The region of the U.S. was determined using the four regions identified by the 2020 U.S. Census Bureau, including Northeast, South, Midwest, and West (Figure 1) [12].

Initially, characteristics of male and female professors with and without endowed chairs were tabulated. Variables were chosen a priori based on conceptual relevance. Logistic regression was used in univariable and multivariable models to compute the odds of chair endowment versus no endowment for levels of all variables compared to their reference categories. For the models, men were compared to women, participants with two titles and three titles were each compared to those with zero or one title, ranked hospitals were compared to unranked, South, Midwest, and West regions were compared to the Northeast, the PhD degree was compared to the MD degree, full professor was compared to all other professional levels combined, and all subspecialties were compared. In addition to these comparisons, all pair-wise comparisons were obtained for multi-level variables through contrasts. P-values less than 0.05 were considered significant. No adjustment of the significance level was made for multiple comparisons. The data analysis for this study was generated using SAS version 9.4 (SAS Institute Inc., Cary, NC, USA).

## Results

A total of 2,262 faculty were identified at 41 academic ophthalmology institutions across the U.S., and 1,402 (62%) men and 860 (38%) women were included in this study (Table 1). Ophthalmologists with DO degrees were excluded from the analysis due to low sample size (n=26), and zero of the physicians with DO degrees had endowed chair status. Of the 2,262 faculty, 246 (10.9%) carried endowed chair titles. Among these 246 faculty, 59 (24%) were women and 187 (76%) were men. In the overall cohort, this corresponds to 2.6% of all faculty being women with endowed chairs, and 8.3% being men with endowed chairs. Among most subgroups, including the number of additional titles, region of the U.S., academic degree, and professor status, men held more endowed chairs than women. Ocular oncology and non-retina uveitis faculty were the only two subspecialties with women having a greater proportion of endowed chairs than men. Each of these two subspecialties had a similar number of men and women faculty: nine (50%) women in ocular oncology and 20 (47%) women in non-retina uveitis.

**Table 1:** Characteristics of Professors With and Without Endowed Chairs The data has been represented as N (%).

Characteristic	Female, N=860 (38.0%)		Male, N=1402 (62%)	
	With Endowed Chairs, N=59 (6.9%)	Without Endowed Chairs, N=801 (93.1%)	With Endowed Chairs, N=187 (13.3%)	Without Endowed Chairs, N=1215 (86.7%)
Region				
Northeast	10 (4.2)	230 (95.8)	32 (8.7)	338 (91.3)
South	21 (10.5)	178 (89.5)	81 (25.6)	235 (74.4)
Midwest	21 (7.6)	256 (92)	49 (10.8)	406 (89.2)
West	7 (4.9)	137 (95)	25 (9.6)	236 (90.4)
Degree				
MD	54 (6.7)	755 (93.3)	158 (12.8)	1072 (87.2)
PhD	5 (9.8)	46 (90.2)	29 (16.9)	143 (83.1)
Professorship Level				
Assistant professor	4 (1.1)	378 (98.9)	6 (1.6)	379 (98.4)
Associate professor	9 (5.5)	155 (94.5)	20 (8.3)	221 (91.7)
Full professor	45 (30.6)	102 (69.4)	155 (33.1)	313 (66.9)
Other	1 (0.6)	166 (99.4)	6 (2.0)	302 (98.0)
Number of Other Titles				
0	21 (3.1)	649 (96.9)	82 (8.0)	943 (92)
1	21 (16.3)	108 (83.7)	53 (21.4)	195 (78.6)
2	14 (29.2)	34 (70.8)	29 (36.2)	51 (63.8)
3	2 (20.0)	8 (80.0)	15 (44.1)	19 (55.9)
4	1 (50)	1 (50)	7 (87.5)	1 (12.5)
5	0	0	1 (50.0)	1 (50.0)
6	0	0	0	2 (100)
7	0	1 (100)	0	1 (100)
9	0	0	0	1 (100)
Ranking Status				
Ranked	25 (7.4)	312 (92.6)	89 (15.5)	485 (84.5)
Unranked	34 (6.5)	489 (93.5)	98 (11.8)	730 (88.2)
Subspecialty				
Comprehensive	5 (2.3)	209 (97.7)	38 (8.5)	408 (91.5)
Glaucoma	9 (7.0)	119 (93.0)	25 (14.4)	148 (85.6)
Pediatric	7 (6.2)	106 (93.8)	11 (12.8)	75 (87.2)
Uveitis/retina	18 (15.0)	102 (85.0)	67 (22.4)	232 (77.6)
Cornea	9 (7.4)	112 (92.6)	23 (11.8)	172 (88.2)
Oculoplastic	2 (3.0)	64 (97.0)	7 (9.3)	68 (90.7)
Neuro ophthalmology	5 (9.6)	47 (90.4)	10 (13.3)	65 (86.7)
Uveitis/nonretinal	1 (5.0)	19 (95.0)	1 (4.4)	22 (95.6)
Pathology	1 (5.9)	16 (94.1)	4 (19.1)	17 (80.9)
Ocular oncology	2 (22.2)	7 (77.8)	1 (11.1)	8 (88.9)

In the univariable model, the odds for an endowed chair were two times greater for males than for females (p<0.001), though gender was not significant in the multivariate model (Table 2). Similarly, odds were 32% greater for those from ranked departments compared to non-ranked in the univariable model (odds ratio (OR)=1.32, confidence interval (CI)=1.01, 1.72). However, this significance disappeared in the multivariable model. Otherwise, the results were similar when variables were considered alone or when combined in one model.

**Table 2:** Odds Ratios and Confidence Intervals From Univariable and Multivariable Models *Due to numerous comparisons between pairs of subspecialities, only those pairs that were significantly different in either the univariable or the multivariable analyses are presented. Odds ratios and 95% confidence intervals are reported. P-values were calculated using the Wald test. P-values less than 0.05 were considered significant (bolded).

Variable	Category	Univariable				Multivariable			
		Odds Ratio	95% Confidence Interval	Z-score	P-value	Odds Ratio	95% Confidence Interval	Z-score	P-value
Gender	Male vs. Female	2.09	1.54, 2.84	4.72	<0.001	1.35	0.94, 1.95	1.61	0.107
Number of titles	2 vs. 0-1	5.42	3.64, 8.07	8.32	<0.001	4.63	2.84, 7.54	6.15	<0.001
	≥ 3 vs. 0-1	7.96	4,68, 13.52	7.67	<0.001	4.92	2.57, 9.42	4.81	<0.001
	≥ 3 vs. 2	1.47	0.78, 2.75	1.20	0.229	1.06	0.05, 2.26	0.06	0.873
Rank	Ranked vs. Unranked	1.32	1.01, 1.72	2.04	0.040	1.37	0.98, 1.90	1.86	0.062
Region	South vs. Northeast	3.34	2.28, 4.89	6.20	<0.001	3.79	2.38, 6.02	5.63	<0.001
	Midwest vs. Northeast	1.43	0.96, 2.13	1.76	0.080	1.59	0.99, 2.55	1.92	0.056
	West vs. Northeast	1.16	0.72, 1.87	0.61	0.542	0.90	0.52, 1.57	-0.37	0.722
	South vs. Midwest	2.34	1.68, 3.25	5.05	<0.001	2.38	1.60, 3.56	4.25	<0.001
	South vs. West	2.88	1.89, 4.39	4.92	<0.001	4.19	2.54, 6.90	5.62	<0.001
	Midwest vs. West	1.23	0.79, 1.90	0.93	0.352	1.75	1.06, 2.91	2.17	0.029
Degree	PhD vs. MD	1.55	1.05, 2.29	2.20	0.028	3.19	1.67, 6.09	3.51	<0.001
Professional Level	Full professor vs. all other levels	16.78	11.97, 23.54	16.35	<0.001	13.21	9.21, 18.94	14.03	<0.001
Subspecialty*	Cornea vs. Comprehensive	1.62	1.00, 2.61	1.97	0.048	2.42	1.22, 4.81	2.53	0.011
	Glaucoma vs. Comprehensive	1.83	1.14, 2.93	2.51	0.012	2.77	1.41, 5.43	2.96	0.003
	Glaucoma vs. Oculoplastic	1.87	0.87, 4.02	1.60	0.109	2.41	1.01, 5.74	1.98	0.047
	Pediatric vs. Comprehensive	1.43	0.80, 2.54	1.21	0.223	2.53	1.17, 5.55	2.34	0.020
	Neuro-ophthalmology vs. Comprehensive	1.92	1.03, 3.58	2.05	0.039	2.72	1.21, 6.10	2.42	0.015
	Uveitis/retina vs. Neuro-ophthalmology	1.90	1.05, 3.42	2.13	0.033	1.78	0.91, 3.47	1.69	0.093
	Uveitis/retina vs. Glaucoma	2.00	1.30, 3.07	3.16	0.002	1.75	1.05, 2.92	2.14	0.032
	Uveitis/retina vs. Cornea	2.26	1.46, 3.50	3.66	<0.001	2.00	1.20, 3.33	2.66	0.008
	Uveitis/retina vs. Pediatric	2.56	1.49, 4.39	3.41	0.001	1.74	1.04, 3.64	1.73	0.049
	Uveitis/retina vs. Comprehensive	3.66	2.48, 5,41	6.52	<0.001	4.84	1.86, 9.52	3.79	<0.001
	Uveitis/retina vs. Oculoplastic	3.73	1.82, 7.63	3.60	<0.001	3.73	1.82, 7.63	3.60	<0.001
	Uveitis/retina vs. Pathology	1.68	0.64, 4.42	1.05	0.295	3.91	1.27, 12.05	2.38	0.017
	Uveitis/retina vs. Uveitis/Nonretinal	5.23	1.24, 22.00	2.26	0.024	5.23	1.24, 22.00	2.26	0.024

Findings from the multivariable analysis indicate that the odds of an endowed chair were greater for those with more than one title. The odds were 4.63 times (CI=2.84, 7.54) greater for those with two titles, and 4.92 (CI=2.57, 9.42) times greater for those with three titles. With respect to region, the odds of endowment for faculty from the South were greater than for those from the three other regions. The odds were nearly four times greater compared to both the Northeast and the West, and more than two times greater compared to the Midwest. Those from the Midwest also had significantly greater odds than those from the West (OR=1.75, p=0.029). The odds of endowment for those with a PhD degree were three times greater than those with an MD degree (OR=3.19, CI=1.67, 6.01). The odds were much greater for full professors than for all other levels combined (OR=13.21, CI=9.21, 18.94). As shown in Table 2, the odds were two to five times greater for those within the uveitis/retina specialty compared to each of the other specialties. Most other specialties had greater odds than those from the comprehensive specialty.

## Discussion

Among 41 academic ophthalmology institutions in the U.S., professor level, the region of the country, graduate degree, ophthalmic subspecialty, and number of additional titles were all found to have a significant independent relationship with whether a faculty member held an endowed chair or not. Specifically, having two or more additional titles, an institution location in the South, a PhD degree, specializing in uveitis/retina, and full professorship all conferred greater odds of endowed chair status. The significantly lower odds of women granted endowed chair status that were present on univariable analysis were no longer significant upon multivariable analysis, suggesting that there are other underlying factors influencing the odds of having an endowed chair.

While gender itself may not have a significant relationship with endowed chair status, men may be more likely than women to achieve many measures of success that are considered when granting endowed chairs. Being the senior author of peer-reviewed publications, for example, is one of the most respected positions within the authorship list. Yet, in 2020, women ophthalmologists who were senior authors, defined as the last author on a peer-reviewed publication, accounted for only 27.1% of publications in the top 30 ophthalmology journals [13]. Another measure of scholarly impact is the H-index, and in early career stages, women have significantly lower H-indices than men [14]. This discrepancy in senior authorship between men and women likely influences the ability of females to earn an endowed chair during their the early- to mid-phases of their careers, given that research productivity and publications are heavily considered in career advancement in academic medicine. During later career stages, women surpass men in scholarly productivity, suggesting that when influences from familial obligations such as childbearing and child-rearing wane, women have the ability to dedicate more time to their academic endeavors [14].

Data published by the Association of American Medical Colleges shows that across all specialties, 28% of all full professors are women [15]. In this study, full professorship was found to have the highest association with endowed chair status (Table 2); however, only 24% of full professors were women. A similar study in oncology did not find gender differences in endowed chair status in full professors but did identify a significant gender disparity in full professorship allocation [7]. This gender difference does not appear to be diminishing over time either. A recent study reported a total increase of 450 women and 350 men ophthalmologists from 2003 to 2017, yet the number of women full professors increased only by 90 compared to 200 for men full professors within the same time frame [16]. This suggests that although women may be entering ophthalmology at a higher rate than men, the gender gap in full professorship is actually widening.

Our study has some inherent limitations. First, it was not possible to identify factors such as scholarly productivity and years of experience for individual faculty members at the time that they may have been considered for an endowed chair. In addition, the differences in endowed chair selection among institutions could not be determined with our study design. It was also not possible to access additional demographic information from online resources about each faculty member, such as race and ethnicity, that may be associated with receiving an endowed chair title. As a cross-sectional study based on publicly available data, we were limited to reporting associations without establishing causality or temporality. Further research with additional comparison groups, including factors such as external funding and years in clinical practice at an academic institution, will facilitate a deeper understanding of factors impacting endowed chair selection. Identification of barriers and inequities for women and other underrepresented groups in receiving endowed chairs can support the development of more equitable and transparent selection processes. By illuminating structural and institutional patterns in endowed chair allocation, this study contributes to broader efforts to improve fairness in academic recognition and leadership. These findings can inform institutional policy reform and promote a more inclusive academic culture, ultimately enhancing diversity of thought and leadership in the scientific community.

## Conclusions

This study found that endowed chair allocation in academic ophthalmology is influenced by multiple independent factors, including academic rank, subspecialty, geographic region, and academic titles. While gender was not independently associated with endowed chair status on multivariable analysis, persistent disparities in academic advancement metrics suggest underlying structural and institutional barriers for women. These findings highlight the need for targeted efforts to improve equity in academic recognition and advancement. Future studies incorporating detailed measures of scholarly productivity and institutional practices should be conducted to inform policy changes and promote equitable allocation of academic honors such as endowed chair status.
